# Predictive factors of operability after neoadjuvant chemotherapy in resectable or borderline resectable pancreatic cancer: a single-center retrospective study

**DOI:** 10.1007/s12672-021-00462-1

**Published:** 2022-01-03

**Authors:** Masatoshi Murakami, Nao Fujimori, Akihisa Ohno, Kazuhide Matsumoto, Katsuhito Teramatsu, Yu Takamatsu, Ayumu Takeno, Takamasa Oono, Toshiya Abe, Noboru Ideno, Naoki Ikenaga, Kohei Nakata, Masafumi Nakamura, Kousei Ishigami, Yoshihiro Ogawa

**Affiliations:** 1 Department of Medicine and Bioregulatory Science, Graduate School of Medical Sciences, Kyushu University, 3-1-1 Maidashi, Higashi-ku, Fukuoka, 812-8582 Japan; 2 Department of Surgery and Oncology, Graduate School of Medical Sciences, Kyushu University, Fukuoka, Japan; 3 Department of Clinical Radiology, Graduate School of Medical Sciences, Kyushu University, Fukuoka, Japan

**Keywords:** Pancreatic neoplasms, Pancreatic cancer, Neoadjuvant chemotherapy, Operability, Recurrence

## Abstract

**Background/Aims:**

Recently neoadjuvant chemotherapy (NAC) for pancreatic cancer has been shown to be superior to upfront surgery, but it remains a matter of debate for resectable cases. In clinical practice, some resectable cases may become unresectable after NAC. This study aimed to reveal the outcomes after NAC and to clarify the characteristics of unresected cases.

**Methods:**

The medical records of 142 patients who underwent NAC between 2016 and 2020 were retrospectively reviewed. Patient characteristics, effectiveness of NAC, and outcomes were compared between the surgical group and non-surgical group (NSG). Furthermore, the risk of recurrence limited to in the patients who received NAC with gemcitabine plus nab-paclitaxel, which were mostly administered in this cohort, following R0/R1 resection was assessed.

**Results:**

The overall and R0 resection rates after NAC were 89.1% and 79.7%, respectively. The neutrophil to lymphocyte ratio (NLR) > 2.78 (p = 0.0120) and anatomical borderline resectable pancreatic cancer (p = 0.0044) revealed a statistically significantly correlation with the NSG. On the other hand, NAC week < 8 (p = 0.0285), radiological response, stable disease or progression disease (p = 0.0212), and pathological stage > IIA (P = 0.0003) were significantly associated with recurrence. The tumor response rate was approximately 26.1%, and three patients with ≥ 30% reduction of primary tumor lost excision opportunities because of metastasis, interstitial pneumonia, and vascular invasion.

**Conclusions:**

This study shows incomplete tumor shrinkage benefits, but pre-NAC NLR is a predictive factor for predicting operability after NAC. The NLR can be easily calculated by normal blood test, and can be considered as a suitable marker of operability.

**Supplementary Information:**

The online version contains supplementary material available at 10.1007/s12672-021-00462-1.

## Introduction

The incidence of pancreatic ductal adenocarcinoma (PDAC) has been increasing; PDAC is the seventh leading cause of cancer-related deaths globally [1]. PDAC remains as one of the most lethal malignancies, with a 5-year survival rate of approximately 8% [2]. Because of its high invasiveness and its associated challenges for early detection, only ~ 20% of patients with PDAC are amenable to resection at diagnosis [3]. Alternatively, radical resection is the necessary treatment, although quite a few recurrences are observed. Adjuvant chemotherapy (AC) was shown to delay the time to recurrence [4–6], and recently, the efficacy of neoadjuvant chemotherapy (NAC), which is expected to control local disease and eliminate occult metastasis before surgery, has been reported [7–10]. Although there is an uncertainty regarding the need for NAC to treat all resectable pancreatic cancers (RPC), NAC is gaining prominence as a close-to-standard strategy for PDAC cases that are considered for surgical resection.

However, occasionally in clinical practice, there are missed opportunities to excise tumors owing to tumor progression or severe adverse events (AEs) of NAC. Moreover, recurrences of PDAC are common despite NAC and AC being performed thoroughly. Therefore, there is an emergent need to scrutinize the predictive factors of operability and postoperative recurrences of PDAC after NAC.

The neutrophil to lymphocyte ratio (NLR), modified Glasgow prognostic score (mGPS), and several other systemic inflammatory markers have been reported as prognostic indicators of recurrence and survival in patients with several cancers, including PDAC [11, 12]. Recently, the relationships between these inflammatory biomarkers and NAC or neoadjuvant chemoradiotherapy (NACRT) in PDAC have been reported. Hasegawa et al. reported the usefulness of pre-treatment NLR in predicting the pathological responses to NACRT in PDAC [13]. Oshima et al. reported that pre-NACRT mGPS and increased C-reactive protein/albumin ratio after NACRT are independent prognostic factors for patients with PDAC who received NACRT following pancreatectomy [14]. However, only a few studies evaluated such inflammatory biomarkers in pancreatic cancer after NAC. Moreover, no report has, until now, described the relationship between systemic inflammatory biomarkers and the operability after NAC.

Thus, this study aimed to clarify the characteristics of unresected PDAC cases in real-world data, determine the relationships between operability and pre-treatment NLR or mGPS, which are two of the most reported systemic inflammatory markers about various cancers, reveal outcomes, and discuss issues of NAC for patients with RPC or borderline resectable pancreatic cancer (BRPC).

## Methods

### Patients and data collection

**Fig. 1:**
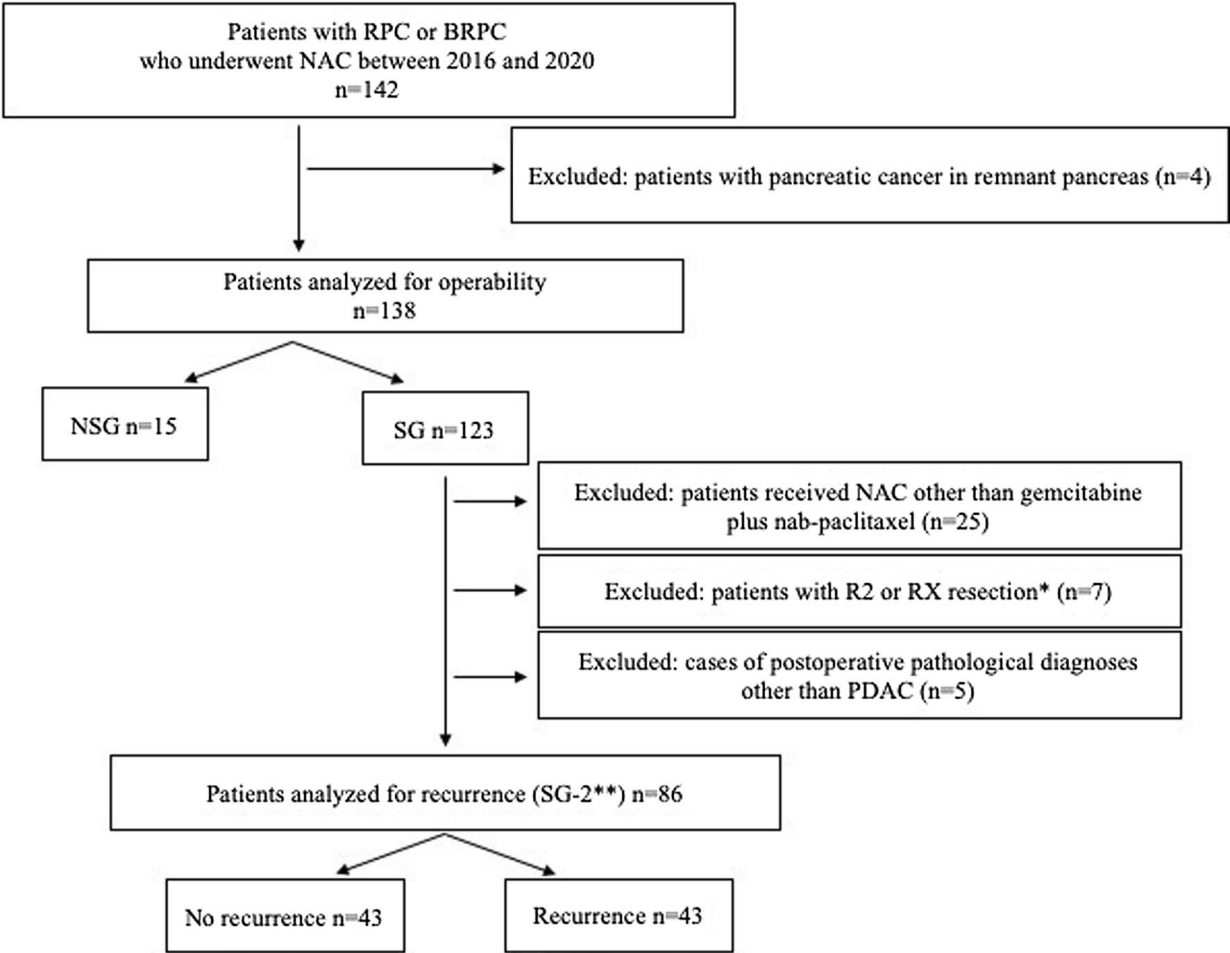
Algorithms for patient inclusion and exclusion in the present study. Total 138 patients and 86 patients were analyzed for operability and recurrence, respectively. *RPC* resectable pancreatic cancer, *BRPC* borderline resectable pancreatic cancer, *NAC* neoadjuvant chemotherapy, *NSG* non-surgical group, *SG* surgical group, *PDAC* pancreatic ductal adenocarcinoma. *R0 microscopic negative margin, *R1* microscopic positive margin, *R2* macroscopic positive margin, *RX* positive margin cannot be assessed. **SG-2: patients with PDAC who received NAC with gemcitabine plus nab-paclitaxel following R0/R1 resection

The medical records of 142 patients with RPC /BRPC who underwent NAC between 2016 and 2020 were collected and retrospectively reviewed. The following exclusion criteria were applied: (1) locally advanced unresectable or metastatic pancreatic cancer at initial diagnosis, (2) age younger than 20 years, (3) non-treatment-naïve patients, (4) other advanced cancer at initial diagnosis, (5) poor surgical candidate at the initial diagnosis, and (6) withdrawal of consent at any time. The analysis of postoperative recurrence was limited only to recurrences after NAC with gemcitabine plus nab-paclitaxel (GnP) after R0/R1 resection in PDAC (defined as surgical group-2 [SG-2], excluding patients who received some other NAC than GnP, with R2/RX resection and other than adenocarcinoma on postoperative pathological diagnosis). In total, 138 and 86 patients were analyzed for operability and recurrence, respectively. Figure 1 shows the patient selection criteria used in this study.

The clinicopathological factors analyzed in all patients were as follows: age, sex, American Society of Anesthesiologists physical status (ASA-PS), diabetes, jaundice, resectability classification according to the National Cancer Network guideline [15], tumor location, tumor size, carbohydrate antigen 19-9 (CA19-9) levels, NLR, mGPS, type of NAC and AC, duration and relative dose intensity (RDI) of NAC, radiological response [16], AEs graded using the Common Terminology Criteria for Adverse Events v5.0, reasons for unresectable tumors, date of diagnosis, surgery and recurrence, surgical procedures, combined vascular resection, resection margin status, lymph node status, perioperative complications, and pre- and postoperative pathological diagnosis. We also analyzed the outcomes (overall survival) between the surgical (SG) and non-surgical groups (NSG), moreover with and without recurrence in the SG-2.

Operability was defined as whether or not the patient underwent surgery after NAC. Therefore, in our study, the SG included inoperable or non-curative resection (R2 resection) cases because of dissemination and/or metastasis detected during surgery (n = 4). Blood examination data and radiological images were obtained within 1 month before and after NAC. In patients with obstructive jaundice, the blood examination data after biliary drainages were primarily used. The cut-off values were determined based on a previous report [17]. The optimal cut-off value of NLR was calculated by receiver operating characteristic curve analysis, and the best cut-off value for operability was 2.78. Staging, resectability classification, and the decision on whether to perform NAC and NAC regimens were determined by consensus among surgeons, radiologists, and gastroenterologists.

This study was approved by the Institutional Review Board of Kyushu University Hospital (2021-36). As this study was a retrospective observational study without any invasion and targeted patients who had visited our hospital in the past, we published the materials on the homepage to disseminate the information of this research and gave them an opportunity to refuse participation (opt-out form).

### Neoadjuvant chemotherapy

The NAC treatments administered included gemcitabine (GEM), GnP, gemcitabine plus tegafur/gimeracil/oteracil (GS), and modified FORFIRINOX (mFFX, ﻿fluorouracil, leucovorin, irinotecan, and oxaliplatin). Each regimen was based on a previously reported protocol [7, 18, 19]. After initiating NAC, contrast-enhanced computed tomography or magnetic resonance imaging was performed every 2–3 months, and pancreatic resection was scheduled if margin-negative resection was considered possible.

### Surgical procedure

Pancreatoduodenectomy, distal pancreatectomy, and total pancreatectomy were performed according to the location of the tumor. Superior mesenteric vein/portal vein resection was performed if necessary, although there was no arterial resection in this study.

### Adjuvant chemotherapy

Of 123 patients who underwent curative resection (R0/R1/RX), 119 received AC. Tegafur/gimeracil/oteracil (S-1), GnP, GEM, and mFFX were administered as AC and continued for more than 6 months unless the tolerability was not acceptable.

### Follow up

Patients were followed up quarterly with imaging for the first 2 years and every 6 months thereafter in the SG. The NSG patients were followed up every 3–4 months during cancer treatment.

### Statistical analyses

﻿Statistical analyses were performed using JMP ver. 12 (SAS Institute Inc.). The Kaplan–Meier plots with log-rank testing were used to analyze the overall survival. Univariate and multivariate analyses using logistic regression models were conducted to identify predictive factors for operability after NAC and assessing the risk of recurrence in the SG-2. Variables with p < 0.1, on univariate analysis, were further included in the multivariate analysis. Statistical significance was set at p < 0.05.

## Results

### Patient characteristics at baseline before NAC and NAC characteristics

Table 1 summarizes the patient characteristics before NAC. This cohort had a relatively large number of BRPC cases (45.7%). The borderline resectable-venous and borderline resectable-arterial (BR-A) cases were 22 and 41 patients, respectively. Forty patients (29.0%) developed diabetes 1 year before the diagnosis of pancreatic cancer. Forty patients had jaundice before the induction of NAC, and all of them improved with biliary drainage. The median NLR was 2.11 (range: 0.66–6.58); and 98, 21, and six patients had mGPS-0, mGPS-1, and mGPS-2, respectively. More than 80% patients received GnP. NAC was reduced or discontinued because of AEs (regarding biliary infections, NAC was reduced only in patients with repeated biliary infections despite biliary drainage or antibiotics) and the median RDI of NAC was 78.0% (33–100%).

**Table 1:** Patient characteristics before neoadjuvant chemotherapy (NAC) and NAC characteristics

Variables	Number
Patient characteristics (n = 138)	
Age, years [median (range)]	69 (34–85)
Sex (male/ female)	74/64
ASA-PS (1/ 2/ 3)	15/114/ 9
BMI, kg/m^2^ [median (range)]	21.9 (15.2–33.3)
Presence of diabetes	62 (45.0%)
Presence of jaundice	40 (28.1%)
Neutrophil to lymphocyte ratio [median (range)]	2.11 (0.66–6.58)
Modified Glasgow prognostic score	
0	98
1	21
2	6
NA	13
Pre-NAC CA19-9 level, U/mL [median (range)]	114.6 (5–7766)
Tumor location, pancreatic head	86 (62.3)
Resectability classification	
Resectable	75
Borderline resectable-venous	22
Borderline resectable-arterial	41
Pre-NAC tumor size, mm [median (range)]	25 (0–68)
Pre-NAC pathological diagnosis	
Adenocarcinoma	125
No evidence of malignancy	11
NA	2
Pathological examinations and diagnosis yield before NAC^*^	
ERCP (n = 93)	62 (66.7)
EUS-FNA/B (n = 76)	65 (85.5)
Endoscopic biopsy (n = 2)	2 (100)
NAC characteristics	
Type of NAC	
Gemcitabine plus nab-paclitaxel	112
Gemcitabine plus tegafur/gimeracil/oteracil	20
Modified FORFIRINOX	5
Gemcitabine	1
Time period of NAC, weak [median (range)]	10 (3–41)
Relative dose index, % [median (range)]	78.0 (33–100)
Greater than or equal to Grade 2 non-hematological AE^**^	
Pulmonary fibrosis/ pneumonitis (intestinal pneumonia)	8 (5.8)
Peripheral neuropathy	4 (2.9)
Erythema multiforme	3 (2.2)
Biliary tract infection	16 (11.6)
Greater than or equal to Grade 3 hematological AE^**^	
Neutrophil count decreased	76 (55.1)
Febrile neutropenia	7 (5.1)
Alanine aminotransferase increased	2 (1.4)
Anemia	1 (0.7)

NAC, neoadjuvant chemotherapy; ASA-PS, American Society of Anesthesiologists physical status; NA, not available; CA 19–9, carbohydrate antigen 19–9; EUS-FNA/B, endoscopic ultrasound-guided fine needle aspiration/biopsy; AE, adverse event

*Overlapping

**Common Terminology Criteria for Adverse Events v5.0

### Post-NAC patient characteristics and postoperative clinicopathological quality

Table 2 shows the clinicopathological characteristics of patients after NAC and surgery. The objective response and disease control rates were 25.9 and 92.6%, respectively. The overall resection and R0 resection rates were 89.1 and 79.7%, respectively. The reasons for not undergoing resection were arterial invasion (nine patients) (progressed to unresectable locally advanced in eight patients and progressed from R- to BR-A in the other patient at the time of re-evaluation after NAC), metastasis (four patients), intestinal pneumonia, and other (one patient each). Three of the 75 patients with RPC could not undergo tumor excisions after NAC owing to vessel invasion or newly emerged paraaortic lymphadenopathy and liver metastases (shown in the representative images of inoperable RPC in Additional file 1: Fig. S1). Compared to the pathological tumor size, post-NAC tumor size on imaging was more than 10 mm underestimated and overestimated in 59 (48.0%) and four patients (3.3%), respectively (data not shown). Pathological lymph node statuses were positive in 76 patients, and only > 30% of them could be predicted by radiation images before NAC. No perioperative mortality occurred in this cohort.

**Table 2:** Post- neoadjuvant chemotherapy patient characteristics and clinicopathological characteristics of the surgery

Variables	Number
Treatment response of NAC (n = 138)	
Post-NAC CA19-9 level, U/mL [median (range)]	29.2 (1.5–1049)
Post-NAC tumor size, mm [median (range)]	20 (0–67)
Radiological response	
Complete response	2
Partial response	33
Stable disease	90
Progressive disease	10
NA	3
Unresectable reasons (n = 15)	
Vessel invasion	9
Metastasis	4
Intestinal pneumonia	1
Undefined	1
Resection rate	123 (89.1)
R0 resection	110
R1 resection	5
R2 resection	4
RX resection	4
Postoperative variables (n = 123)	
Presence of PV/SMV resection	30 (24.4)
POPF by ISGPS, grade B or C	18 (14.6)
Clavien-Dindo grade > 2	37 (26.8)
Postoperative tumor size, mm [median (range)]	28 (8–83)
Presence of pathological lymph node metastasis	76 (61.8%)
Postoperative pathological diagnosis	
Adenocarcinoma	115
Adenosquamous carcinoma	4
Invasive intraductal papillary mucinous carcinoma	1
Mixed acinar-neuroendocrine carcinoma	1
Mixed neuroendocrine non-neuroendocrine neoplasm	1
Neuroendocrine tumor (G3)	1
Adjuvant chemotherapy (n = 119*)	
Tegafur/gimeracil/oteracil	100
Other	4
None	15

*NAC* neoadjuvant chemotherapy; CA 19-9, carbohydrate antigen 19-9, *NA* not available, *R0* microscopic negative margin, *R1* microscopic positive margin, *R2* macroscopic positive margin, *RX* positive margin cannot be assessed, *PV/SMV* portal vein/ superior mesenteric vein, *POPF* postoperative pancreatic fistula, *ISGPS* International Study. Group on Pancreatic Surgery

*4 patients (R2 resection) were excluded

### Preoperative and postoperative pathological diagnoses

Of 138 patients who underwent pathological examinations before the induction of NAC, 125 were diagnosed with adenocarcinoma by endoscopic ultrasound-guided fine-needle aspiration/biopsy (EUS-FNA/B), endoscopic retrograde cholangiopancreatography (ERCP), and/or endoscopic biopsy (Table 1). Eleven patients (7.9%) underwent NAC without a pathological diagnosis.

The postoperative pathological diagnosis was adenocarcinoma in 115 patients and other than adenocarcinoma in eight patients (6.5%). Above all, seven patients of them, except those with adenocarcinoma, were preoperatively misdiagnosed as having adenocarcinoma and for the eighth patient, malignancy could not be precluded despite multiple inspections (Table 2).

### Outcomes of NAC

**Fig. 2:**
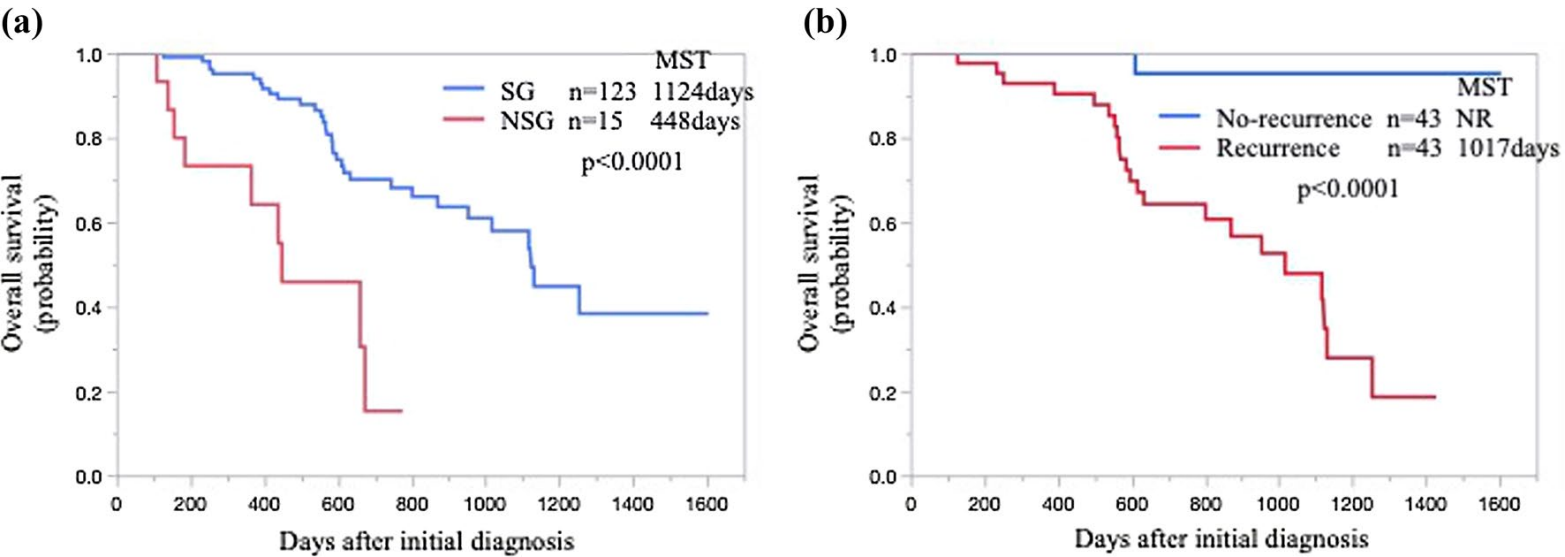
Kaplan–Meier survival curves of patients. **a** Overall survival of all patients in the surgical and non-surgical group. **b** Overall survival of patients with and without recurrences in the SG-2^*^. **a**, **b** The significant differences were analyzed by log-rank tests. *MST* Median survival time; *NR* not reached, *NSG* non-surgical group, *SG* surgical group. *SG-2: patients with pancreatic ductal adenocarcinoma who received neoadjuvant chemotherapy with gemcitabine plus nab-paclitaxel following R0/R1 resection

Figure 2 shows the results of the Kaplan–Meier survival analysis of this cohort. The median survival time was significantly longer for patients in the SG than for those in the NSG (1124 vs. 448 days, p < 0.0001, Fig. 2a). In the SG-2 (n = 86), 43 patients (50.0%) had recurrences, and patients without recurrence had the best prognosis (not reached vs. 1017 days, with and without recurrence in the SG-2, respectively Fig. 2b).

### Predictive factors of resection

First, we analyzed the factors associated with operability after NAC. Based on univariate analysis, seven variables (p < 0.1) were extracted for multivariable analysis (patient age ≥ 65 years, jaundice, NLR > 2.78, mGPS score: 1, 2, tumor size > 20 mm, CA19-9 level > 500 U/mL, anatomical borderline resectable) (Table 3). From the multivariate analysis, NLR > 2.78 (p = 0.0120) and anatomical borderline resectable (p = 0.0044) were revealed as significantly correlated with the NSG.

**Table 3:** Univariate and multivariate analyses of predictive factors associated with operability after neoadjuvant chemotherapy

Factors	SG (n = 123)	NSG (n = 15)	Univariate analysis	Multivariate analysis
Age ≥ 65 years	80 (65.0)	14 (93.3)	0.0127	0.0905
Sex, male	68 (55.3)	8 (53.3)	0.8861	
ASA-PS > 2	9 (7.3)	0 (0)	0.1429	
BMI ≥ 25 or < 18.5 kg/m^2^	91 (74.0)	11 (78.6)	0.7044	
Presence of diabetes	58 (47.2)	5 (33.3)	0.3049	
Presence of jaundice	30 (24.4)	8 (57.1)	0.0143	0.9402
Neutrophil to lymphocyte ratio > 2.78	19 (17.4)	9 (64.3)	0.0003	0.0120
Modified Glasgow prognostic score > 0	21 (18.9)	6 (42.9)	0.0561	0.1723
Tumor size > 20 mm	90 (73.2)	14 (93.3)	0.0549	0.7331
CA19-9 level > 500 U/mL	14 (11.6)	6 (40.0)	0.0097	0.0847
Tumor location, pancreatic head	71 (57.7)	12 (80.0)	0.1005	
Resectability classification, borderline resectable	49 (39.8)	12 (80.0)	0.0026	0.0044
Type of NAC, gemcitabine plus nab-paclitaxel	98 (79.7)	14 (93.3)	0.1717	
Relative dose index < 80%	58 (47.5)	9 (69.2)	0.1479	
Radiological response, < 30% reduction of primary tumor	32 (26.7)	3 (21.4)	0.6670	

*SG* surgical group, *NSG* non-surgical group, *ASA-PS* American Society of Anesthesiologists physical status, *NAC* neoadjuvant chemotherapy, *CA 19-9* carbohydrate antigen 19-9

In these analyses, clinical factors before the induction of NAC are adopted

### Predictive factors of recurrence (among the SG-2)

Next, we reviewed the details of recurrence after surgery because postoperative recurrence was related to prognosis. Despite several types of NAC being administered, more than 80% patients in our cohort received GnP. Moreover, patients with other than adenocarcinoma in the postoperative pathological diagnosis and R2/RX resection were included in the SG group. To avoid all possible biases, we extracted data of only patients with PDAC who received GnP following R0/R1 resection (SG-2). Based on univariate analysis, five variables (P < 0.1) were extracted for multivariable analysis (NAC week < 8, radiological response, stable disease (SD) or progressive disease (PD), pathological stage > IIA, microscopic margin status, non-administration of AC) (Table 4). In the multivariate analysis, NAC week < 8 (p = 0.0285), radiological response, SD or PD (p = 0.0302), and pathological stage > IIA (0.0003) were significantly associated with recurrence.

**Table 4:** Univariate and multivariate analyses of predictive factors associated with postoperative recurrences in the SG-2

Factors	No-Recurrence (n = 43)	Recurrence (n = 43)	Univariate analysis	Multivariate analysis
Age ≥ 65 years	32 (74.4)	30 (69.8)	0.6305	
Sex, male	25 (58.1)	24 (55.8)	0.8276	
ASA-PS > 2	4 (9.3)	2 (4.7)	0.3930	
BMI ≥ 25 or < 18.5 kg/m^2^	10 (23.3)	8 (18.6)	0.5957	
Presence of diabetes	19 (44.2)	25 (58.1)	0.1948	
Presence of jaundice	9 (20.9)	12 (27.9)	0.3807	
Neutrophil to lymphocyte ratio > 2.78	6 (17.1)	10 (25.0)	0.4048	
Modified Glasgow prognostic score > 0	5 (13.9)	8 (20.0)	0.4778	
Pre-NAC CA19-9 level > 500 U/mL	5 (11.9)	4 (9.5)	0.7240	
Tumor location, pancreatic head	25 (58.1)	30 (69.8)	0.2607	
Resectability classification, borderline resectable	18 (41.9)	24 (55.8)	0.1948	
NAC week < 8	1 (2.3)	10 (23.3)	0.0019	0.0285
Relative dose index < 80%	21 (48.8)	16 (37.2)	0.2756	
Radiological response, SD or PD	24 (57.1)	34 (81.0)	0.0172	0.0302
PV/SMV resection	9 (20.9)	15 (34.9)	0.1475	
POPF by ISGPS, grade B or C	7 (16.3)	6 (14.0)	0.7633	
Clavien-Dindo grade > 2	14 (32.6)	15 (34.9)	0.8196	
Pathological stage > IIA	17 (39.5)	34 (79.1)	0.0001	0.0003
Margin status R1	0 (0.0)	3 (7.0)	0.0389	0.0755
Non-administration of adjuvant chemotherapy	1 (2.4)	5 (11.9)	0.0825	0.3003

*ASA-PS* American Society of Anesthesiologists physical status, *NAC* neoadjuvant chemotherapy, *SD* stable disease, *PD* progressive disease, *PV/ SMV* portal vein/ superior mesenteric vein, *POPF* postoperative pancreatic fistula, *ISGPS* International Study Group on Pancreatic Surgery; R1: microscopic positive margin; CA 19–9, carbohydrate antigen 19–9

SG-2: patients with PDAC who received NAC with gemcitabine plus nab-paclitaxel following R0/R1 resection

### Degree of tumor shrinkage in patients with NAC

**Fig. 3:**
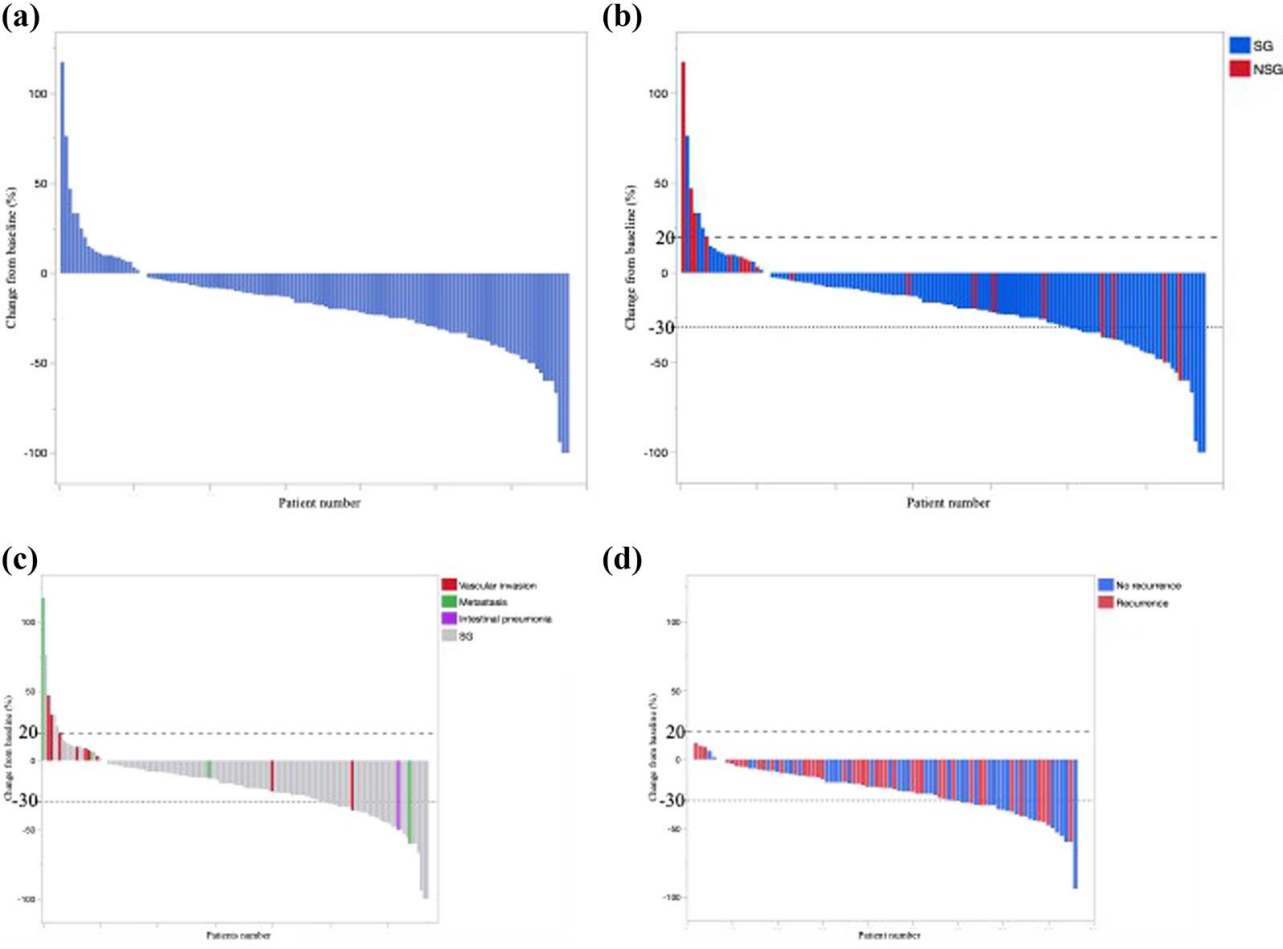
Waterfall plot of tumor response for each patient with imaging performed before and after neoadjuvant chemotherapy (NAC) (n = 135). **a** Total 112 patients (83.0%) experienced tumor shrinkage. **b** Relationship between tumor response and operability after NAC. One-third of the patients in the non-surgical group experienced tumor shrinkage. **c** Relationship between tumor response and unresectable reasons. Importantly, metastases developed even in patients with shrinkage of the primary tumor by NAC. **d** Relationship between tumor responses and recurrences in the SG-2^*^. Recurrence is associated with tumor shrinkage after NAC. *NAC* neoadjuvant chemotherapy, *SG* surgical group, *NSG* non-surgical group. *SG-2: patients with pancreatic ductal adenocarcinoma received NAC with gemcitabine plus nab-paclitaxel following R0/R1 resection

Figure 3a shows a waterfall plot of the tumor shrinkage of the primary lesions by NAC. Total 121 patients showed tumor shrinkage after NAC. The tumor response rate, containing two complete responses and 33 partial responses, was approximately 25.9% (Table 2). Although the reasons for not undergoing resections were vessel invasion, metastasis, interstitial pneumonia, and undefined (Table 2), the operability after NAC was not associated with tumor shrinkage effects (Fig. 3b). Importantly, three patients with ≥ 30% reduction of primary tumor missed their excision opportunities because they developed metastasis, interstitial pneumonia, or vascular invasion even though their primary tumor had shrunk after treatment by NAC (Fig. 3c). On the other hand, in the SG-2, recurrence was related to tumor shrinkage of NAC, although there were some recurrences even with remarkable tumor shrinkage by NAC (Fig. 3d).

## Discussion

This study presents the retrospective review of the data of patients with RPC or BRPC who received NAC at a single center. Further, we evaluated the outcomes and prognosis of NAC. The main finding of our study was that the pre-NAC NLR, but not radiological response, was related to the operability after NAC. Naturally, inoperability after NAC reflects the insufficiency of the tumor shrinkage effect, but the radiological response is often insufficient for predicting the effect of NAC and the operability because tumor size is affected by the inflammation caused by anticancer drugs [20]. In our cases, metastases developed regardless of the shrinkage of the primary site by NAC (Fig. 3c). Therefore, operability is determined not only by the local tumor effect due to local vascular infiltration, but also is affected by other predictive factors, such as the NLR. To our knowledge, this is the first report on the correlation between pre-treatment NLR and operability after NAC. Hence, we propose the pre-treatment NLR as a suitable marker of operability.

Nevertheless, the operability was non-concordant with the response to NAC, and more indices are required to discriminate between the effects of NAC and recurrence. Contrary to previous reports, instead of the high pre-NAC mGPS [14], insufficient local control effect and insufficient time of NAC were related to recurrence and poor prognosis in patients with pancreatic cancer who received NAC following pancreatectomy in this study. Although we have not compared GnP with other regimens about recurrences because a few patients received them, a regimen with a higher response rate has a possibility to improve outcomes after NAC followed by curative resection. In addition, a longer duration of NAC to shrink a tumor enough may be effective, especially in patients with advanced cancer. It has been reported that NAC or NACRT (NA(C)RT) is associated with lower overall recurrence rates, especially liver metastases, and improved locoregional disease control [21] and that NA(C)RT can provide survival benefit in BRPC and a subgroup of RPC patients, but no statistically significant differences were observed in the disease-free survival time and recurrence between the NA(C)RT cases and those that underwent surgical resection first [22]. Preoperative high-risk features, such as CA19-9, lymph node disease, pain, and weight loss, and large size, which were defined in the National Comprehensive Cancer Network or American Society of Clinical Oncology guideline, could indicate the usefulness of NAC by the clear-cut parameters. PDAC could be managed better if the most effective/specific regimen for each case would be clarified in advance. In PDAC, a small number of biomarkers directly linked to treatments have been reported until now [23–26]. There is still room for debate on an optimal period of NAC, where the duration of NAC would predict the risk of recurrence to quantize the respective results of this study. The precise type and duration of NAC will become an issue in the future.

Although this retrospective cohort had a large number of BRPC cases, especially BR-A, the resection rate was relatively good. NAC effectiveness was shown by the high R0 resection rates for this study, but relatively many AEs were observed. Among those, intestinal pneumonia occurred in eight patients and one patient missed the resection opportunity. Outcomes of the NSG are generally unfavorable as treatment options are limited by the ineffectiveness/resistance of NAC regimens, and AE, such as interstitial pneumonia. In addition, the insufficient period of NAC, mainly due to AEs, may be relevant to the poor prognosis because of postoperative recurrences (Table 4). Currently, predicting the outcomes of NAC and the occurrence of AEs is difficult. Sufficient explanations regarding the conditions of patients surrounding NAC, close relationships between physicians and surgeons, sufficient provisions against side effects, and changing therapeutic measures for the clinical situations are essential for better prognosis in such patients.

In the present study 10 patients (7.0%) underwent NAC without pathological diagnosis despite having undergone inspections (in addition, one more patient received NAC without an inspection). EUS-FNA is used for the cytological and histological diagnosis of PDAC. Compared to low complications and mortalities, high sensitivity and specificity for the diagnosis of PDAC have been reported [27, 28]. However, if complications such as pancreatitis occur, or those appliable to ERCP, NAC administration is delayed and the schedule for resection is occasionally missed because of the complications and immune weakness. Regarding EUS-FNA, needle tract seeding cannot be ignored [29, 30]. Multiple inspections increase such complications; thus, it is a dilemma whether to examine until the pathological diagnosis is obtained. Moreover, particular types of pancreatic cancer, such as adenosquamous carcinoma or mixed neuroendocrine non-neuroendocrine neoplasm, in which the efficacy of NAC is not obvious, are sometimes misdiagnosed as adenocarcinoma by EUS-FNA or ERCP. Indeed, some patients were misdiagnosed with adenocarcinoma preoperatively, and NAC was introduced in the present study. The pathological diagnosis before the induction of NAC is obviously, but it still has those limitations.

Our study has several limitations. First, this was a retrospective, single-center study with few patients in the SG and NSG after NAC. Second, the regimen and period of NAC were varied. Although many patients received a couple of NAC cycles, unless the level of unacceptable toxicity was reached, some patients received NAC for relatively long durations until the tumors continued to shrink and until margin-negative resection was deemed possible. Third, although the histological type of pancreatic cancer is heterogenous, operability has been analyzed in patients who received NAC. Therefore, patients whose postoperative pathological diagnoses were not PDAC were included in the analysis of the operability after NAC. Fourth, the number of patients is relatively small (n = 86) and the follow-up time is short in the SG-2 group. Despite the above limitations, our study, reflecting real-world data, produced important clinical values. Nevertheless, a multicenter study with a large series would be able to overcome the challenges of this study.

In conclusion, our study revealed the characteristics of patients with PDAC whose tumors were not resected after NAC followed by the outcomes analysis and further evaluates the challenges of NAC treatment at one Japanese reference hospital. We propose the use of the pre-NAC NLR for predicting the operability after NAC.

## Supplementary Information

**Additional file 1: Figure S1.** The radiological images of a representative resectable pancreatic cancer patient with high pre-neutrophil to lymphocyte ratio (> 2.78) who could not undergo surgical resection after neoadjuvant chemotherapy (NAC). Contrast-enhanced computed tomography images showing pancreatic head cancer **a** before and **b** after NAC. Although the primary lesion remained unchanged after NAC, paraaortic lymphadenopathy (**b**, arrowhead) and **c** a liver metastasis newly appeared on contrast-enhanced magnetic resonance imaging (**c**, arrow).

## Data Availability

All data generated or analyzed during this study are included in this published article.
