# Supplementary figures and images for: Predictive factors of operability after neoadjuvant chemotherapy in resectable or borderline resectable pancreatic cancer: a single-center retrospective study
Source: Discov Oncol. 2022 Jan 3;13:2. doi: 10.1007/s12672-021-00462-1 (PMC8777497; doi:10.1007/s12672-021-00462-1)

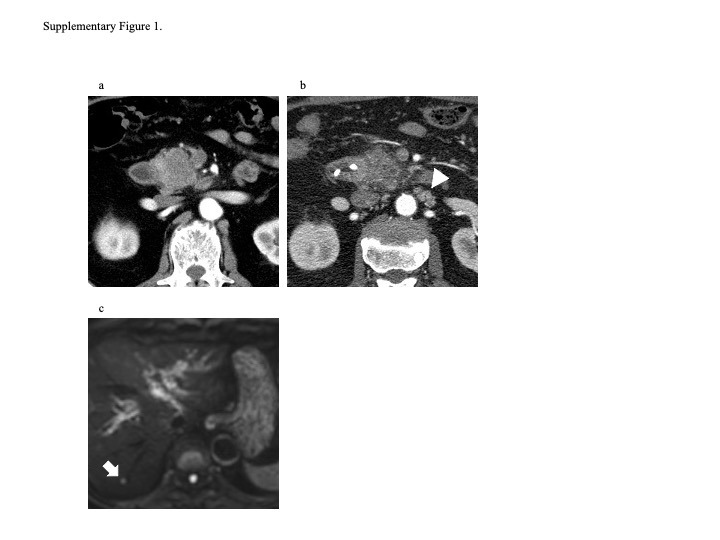

Supplement: Supplementary file 1 — Additional file 1: Figure S1. The radiological images of a representative resectable pancreatic cancer patient with high pre-neutrophil to lymphocyte ratio (> 2.78) who could not undergo surgical resection after neoadjuvant chemotherapy (NAC). Contrast-enhanced computed tomography images showing pancreatic head cancer a before and b after NAC. Although the primary lesion remained unchanged after NAC, paraaortic lymphadenopathy (b, arrowhead) and c a liver metastasis newly appeared on contrast-enhanced magnetic resonance imaging (c, arrow). [file 12672_2021_462_MOESM1_ESM.jpg]
